# Supplementary material for: Timing and origin of natural gas accumulation in the Siljan impact structure, Sweden
Source: Nat Commun. 2019 Oct 18;10:4736. doi: 10.1038/s41467-019-12728-y (PMC6802084; doi:10.1038/s41467-019-12728-y)
Supplement: Supplementary file 2 — Supplementary Information [file 41467_2019_12728_MOESM2_ESM.pdf]

Supplementary Information

“Timing and origin of natural gas accumulation in the Siljan impact structure, Sweden”

Drake et al.

Supplementary note 1. Extended discussion of biodegradation of thermogenic gas, bitumen and oils in the fracture system.

Additional biodegradation signatures interpreted from gas compositions

The lack of ^{13}C-enrichment in C_4 compared to C_3 can be due to that propane is biodegraded most rapidly of the hydrocarbons, already at slight levels of biodegradation[1]. *n*-alkanes are preferentially utilized during biodegradation of oil and gas[2]. The high *i*-C_4/*n*-C_4 in the Siljan gas (Supplementary Data 8, as well as higher *i*-C_5 than *n*-C_5 [below detection]) also suggests significant microbial utilization of primary thermogenic gas, and analogously does high *neo*-C_5/*i*-C_5, because neo-C_5 is relatively resistant to biodegradation[3].

Biomarkers in calcite, bitumen and oils

Bimodal *n*-alkane distribution indicates that the Solberga bitumen formed by mixing of more than one charge of oil at various degrees of degradation[4] suggesting mobilization and degradation of hydrocarbons at several events. Furthermore, presence of a large hump of unresolved complex mixture (UCM) of hydrocarbons in seep oil and bitumen and preferential removal of almost all the *n*-alkanes and alkylcyclohexanes in seep oil are indicative of moderate to severe biodegradation of these materials in the limestone[4]. The irregular *n*-alkane distribution in combination with humps of UCM in the granite fractures (CC1:539; 608) indicate more severe biodegradation of organic matter (Fig. 7). Poor straight chain carbons and S&R-hopanoid isomers in hydrocarbons of the ^{13}C-rich calcite coatings in granite indicate moderate to severe biodegradation. However, the lack of other carbon sources in the granite fractures lead to higher degree of microbial utilization of bitumen and oil stains in the granite fractures (Fig. 7). This is in line with calculated carbon preference index values indicate influence for migrated seep oil/bitumen similar to Bitumen 1 [ref [4]], also deep within the granite (Fig. 7, Supplementary Data 7). These levels of degradation are corroborated by the very small amounts of C_{29} 25-norhopane in the seep oils and bitumen[4].

Supplementary note 2. Summary and interpretation of previous gas data from deep Siljan wells.

Previous investigations in deep boreholes within the central granite dome reported isotopically heavier methane ($\delta^{13}C_{CH4}$: -35 to -15‰ in the superdeep Gravberg-1 well) interpreted as abiotic or thermogenic[5]. Significantly isotopically lighter methane dissolved in groundwater ($\delta^{13}C_{CH4}$ values of -77.9 and -77.6‰) at shallower depth (173-184 m and 443 m), were observed in two other wells in the central dome[6]. ^{13}C-depletion of such magnitude points to microbial methanogenesis. Methane dissolved in groundwater of mixed origin in a 500 m borehole at Solberga that penetrated both sediments and deeper granitic basement had two distinct $\delta^{13}C_{CH4}$ populations: -79‰ and -35‰, interpreted as microbial and thermogenic[6], respectively, marking contribution of gases of different origin to the aquifer.

Supplementary note 3. Potential microbial processes linked to the preserved fatty acids

The preserved fatty acids *n*-C_{12} to *n*-C_{18}, particularly the odd chain and branched iC_{15}, aiC_{15}, *n*-C_{15}, 12Me-C_{16}, aiC_{17}, and 12OH-C_{18} as well as the *n*-alcohols and the 1-o-n-hexadecylglycerol preserved within ^{13}C-rich, methanogenesis-related, calcite coatings can be tied to fermentation[7] and/or sulfate reduction by bacteria[8], in line with the S isotope record in pyrite in the fractures. Even though methanogenesis is commonly attributed to *archaea*, which do not produce phospholipid fatty acids, recent studies highlight bacterial methane production through N-fixation together with CO_2[9], which also is a plausible process in the fractures. Furthermore, soil-derived fungi are able to produce methane through biodegradation in relationship with methanogens[10], but also without[11], and high diversity of fungi have been detected in the continental crust[12,13]. Fungi produce phospholipids and other FA than bacteria, but potential presence of fungi at Siljan is yet unexplored. The particular FA detected cannot be used as diagnostic markers for methanogens, in contrast to the heavy $\delta^{13}C_{calcite}$-values.

Supplementary note 4: Influence of limestone derived DIC and micro-scale isotope distillation

The limestone in VM-1 and Solberga-1 has δ^{13}C values of 0 to+2‰[14] and shallow wells in the aquifer contain HCO_3^- concentrations of up to 400 mg L^{-1}, compared to 10-80 mg L^{-1} in the granitic rock aquifer[6]. In the limestone aquifer, it is more likely that equilibrium between DIC and the wall rock dominates. Methanogenesis through carbonate reduction of limestone derived DIC would require smaller ^{13}C enrichment than utilization of ^{13}C-poor DIC formed by oxidation of organic matter to reach the heavy $\delta^{13}C_{calcite}$ values observed. This means that microbial carbonate reduction may to some degree have utilized limestone derived DIC, at least in the limestone aquifer. However, a system with abundant DIC would likely dilute and mask methanogenesis-related δ^{13}C signatures in the produced carbonates. Nevertheless, there is evidently substantial $^{13}C_{calcite}$ and $^{13}C_{CO2}$ enrichment in the sedimentary aquifer. It has been shown in deep energy-poor fracture systems that isotopic fractionation and distillation can occur in microscale in biofilms resulting in isotopic compositions of produced minerals that are very different from the bulk groundwater. At Äspö in Sweden, pyrite precipitated over a 17yr period from a deep sulfate-rich water with relatively constant $\delta^{34}S_{sulfate}$ of 20-30‰ had δ^{34}S values of -47.3 to +53.3‰[15] and at nearby sites, calcite had extreme δ^{13}C variation (-125 to +37‰) compared to the corresponding deep groundwater $\delta^{13}C_{DIC}$ (-17±3‰)[16]. We propose that similar kinetic microbial processes have locally influenced the DIC signature in the Siljan aquifer, particularly in pore space infiltrated by gases, bitumen and seep oils, as shown by spatial relation of these features to significantly ^{13}C-rich calcite (Fig. 3).

Supplementary note 5. Additional discussion of isotopic compositions of the minerals

Moderately ^{13}C-depleted calcite

The $\delta^{13}C_{calcite}$ values of c. -39 to -35‰ at 175 m depth in the crystalline bedrock is proposed to reflect AOM. These $\delta^{13}C_{calcite}$ values overlap with methane of abiotic, thermogenic and microbial origin[17-19] and dilution by other C sources may have occurred. Moderately depleted $\delta^{13}C_{calcite}$ values of around -30 to -20‰ are overlapping with values of abiotic methane, but can, nevertheless, not be fully distinguished from bicarbonate formed following microbial utilization of seep oils with $\delta^{13}C$ of -30‰[20].

^{34}S-enriched pyrite

During a closed system Rayleigh fractionation cycle the $\delta^{34}S_{SO4}$ values will increase gradually as the sulfate pool is exhausted and consequently pyrite will show a large span in $\delta^{34}S_{pyrite}$ values and increase from core to rim of the crystals[21]. The large $\delta^{34}S_{pyrite}$ variability spanning as much as almost 120‰ overall in the granite fractures (from -41.9‰ to +78.0‰, Supplementary Figure 1d) marks these MSR-related reservoir effects in the fracture system (Supplementary Figure 5b).

Supplementary note 6. Tectonic context of the fracture reactivation events.

The significantly higher $^{87}Sr/^{86}Sr$ values of the Late Cretaceous to Neogene calcite compared to the Late Neoproterozoic-Early Paleozoic calcite (Fig. 2) are proposed to result from increased ^{87}Sr with time due to beta decay of ^{87}Rb in the rocks, and prolonged water rock interaction. This increase is, as expected, of largest magnitude in calcite lining Rb-rich granitic wall rock. The higher $^{87}Sr/^{86}Sr$ of the calcite overgrowths is a relative timing indicator that is in agreement with the U-Pb dating of two temporally separated calcite populations. Although there are a few examples of thin slickenfibre calcite that may be formed during strike-slip movement (e.g. Fig. 2i), the euhedral overgrowths of methane-related calcite on Late Neoproterozoic-Early Paleozoic precursors in open fractures suggest predominant formation in an extensional stress regime. Whether the re-activation of the fracture system at Siljan can be related to tectonic events in the far-field requires temporal linkage to such events. Far-field candidates include: Alpine/Pyrenean crustal shortening documented from calcite formation in faults on the British Isles (55 to 25 Ma[22]), in the Helvetic Alps (35–20 Ma)[23] and in the Early Ordovician platformal limestone at Öland, Sweden (65–55 Ma[24]); northern Europe graben system formation and major fluid circulations (35–30 Ma[25]); opening of the North Atlantic[26]; and Late Cretaceous-Sybhercynian tectonics/faulting in NW Europe[27]. After the North-East Atlantic breakup at the Paleocene–Eocene transition, three phases of uplift by erosion of Upper Cretaceous to Oligocene sediments affected the western Fennoscandian shield[28], that may have induced extensional fracture reactivation in the basement. In summary, there are tectonic events in the far-field and uplift events that temporally coincide with the ages of the methane related calcite at Siljan. Methane cycling can thus be related to these fracture reactivation events which are more than 300 Myr younger than the impact, for which $^{40}Ar/^{39}Ar$ laser dating of melt breccia discloses a late Devonian age (380.9±4.6 Ma[29]).

Supplementary Figures,

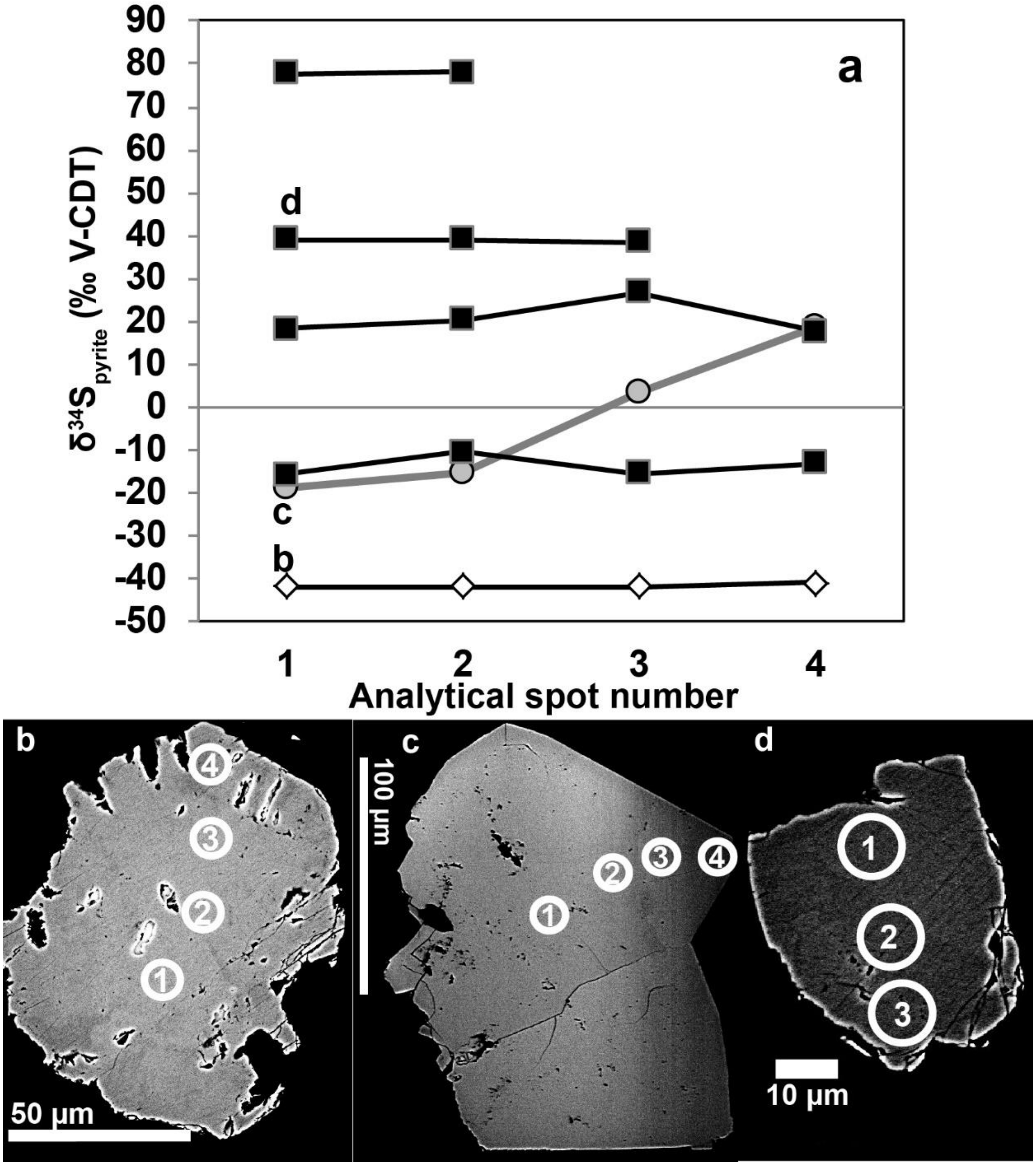

Supplementary Figure 1 $\delta^{34}S$ composition in pyrite crystals. (**a**) Distribution of $\delta^{34}S$ values within selected pyrite crystals. Spot locations are shown in (**b**) CC1:539 m, evenly distributed ^{34}S -depleted values, (**c**) VM2:383 (in paragenesis with ^{13}C-depleted calcite), with increasing $\delta^{34}S$ values with growth; from -19‰ in the interior of the crystal to +19‰ in the rim, (**d**) VM2:642, relatively small variability with crystal growth but overall large variability (-16 to +78‰) of $\delta^{34}S_{pyrite}$ values in other grains in the single fracture (other markers of same type). Errors (2σ) are within the size of the symbols.

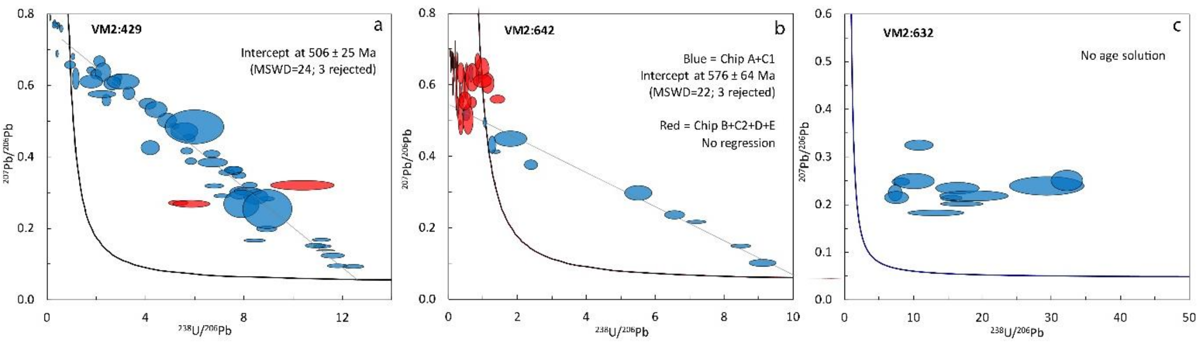

Supplementary Figure 2 U-Pb dating of calcite samples without methane-relation. (**a**) VM2:429, age 506±25Ma. (**b**) VM2:642, age 576±64 Ma. (**c**) VM2:632, no age solution. Errors represented by the ellipses are 2σ.

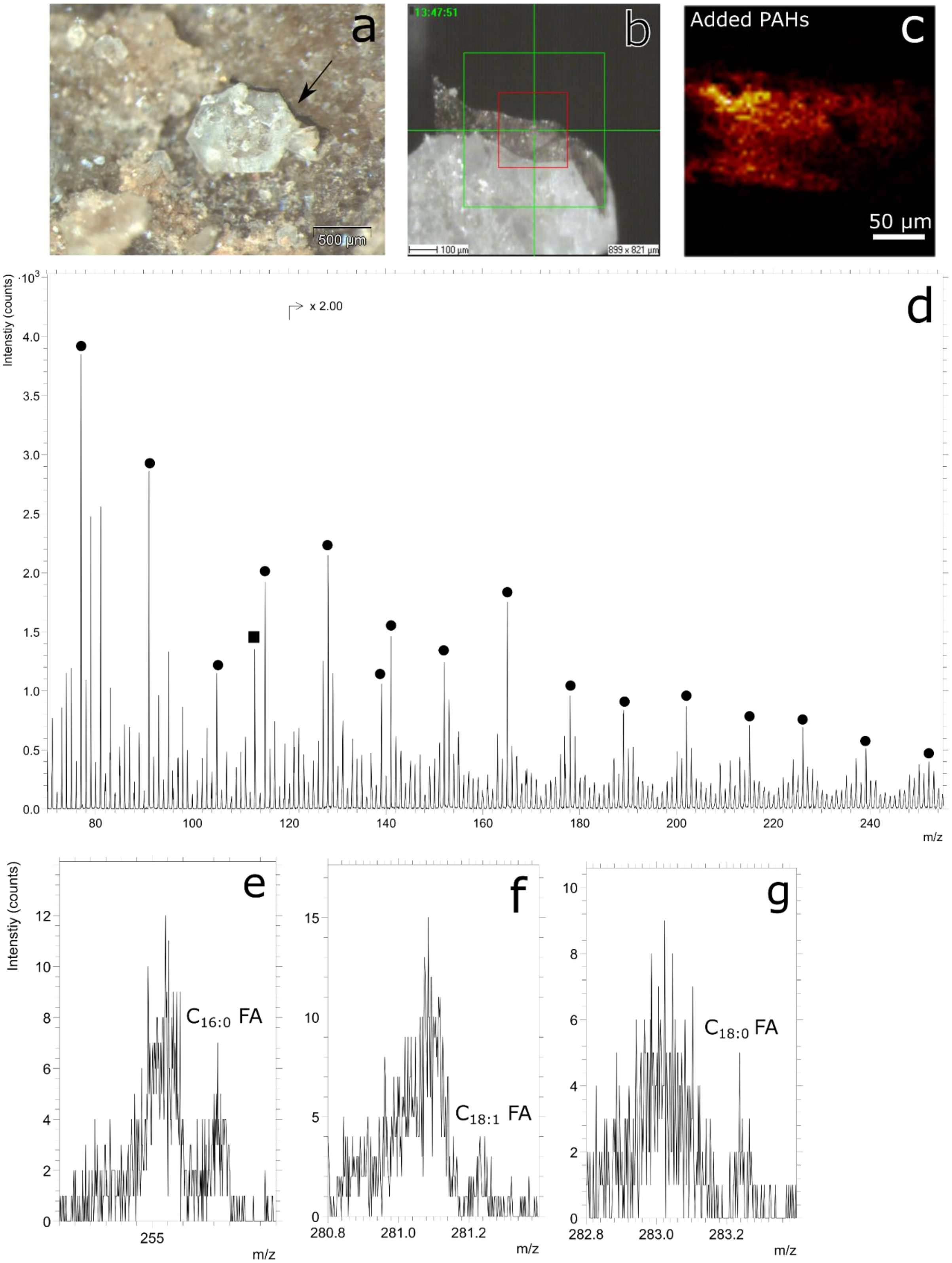

Supplementary Figure 3. ToF-SIMS spectra and ion images of putative organic material. (**a**) Micrograph of area in freshly opened granite fracture of sample CC1:537. Arrow indicates putative organic material on a carbonate crystal. (**b**) ToF-SIMS video of putative organic material on a carbonate crystal. Red square indicates area of ToF-SIMS analysis. (**c**) Combined positive ion image of PAHs at m/z 77.04 (C_6H_5) 91.05 (C_7H_7), 115.05 (C_9H_7), 128.05 ($C_{10}H_8$), 139.05 ($C_{11}H_7$), 141.06 ($C_{11}H_9$), 152.05 ($C_{12}H_8$), 165.06 ($C_{13}H_9$), 178.06 ($C_{14}H_{10}$), 189.05 ($C_{15}H_9$) and 202.06 ($C_{16}H_{10}$). (**d**) Positive ToF-SIMS spectra (m/z 70-255).

Peaks marked by filled circles are assigned to PAHs (77.04, 91.05, 105.07, 115.05, 128.05, 139.05, 141.06, 152.05, 165.06, 178.06, 189.05, 202.06, 215.07, 226.06, 239.06 and 250.05) and one peak marked with square is assigned to an inorganic ion at m/z 112.92 (Ca_2O_2H). Negative ToF-SIMS spectra of (**e**) $C_{16:0}$ fatty acid (m/z 255.2), (**f**) $C_{18:1}$ fatty acid (m/z 281.2) and (**g**) $C_{18:0}$ fatty acid (m/z 283.2). A second type of PAH detected in the sample, of probable modern origin, is shown in Supplementary Figure 4. Circulation of hydrocarbons of sedimentary origin in the deep granitic fracture system is thus also supported by the detected PAH (Fig. 7) which resemble PAH from seep oil in the sediments cropping out at Solberga[30].

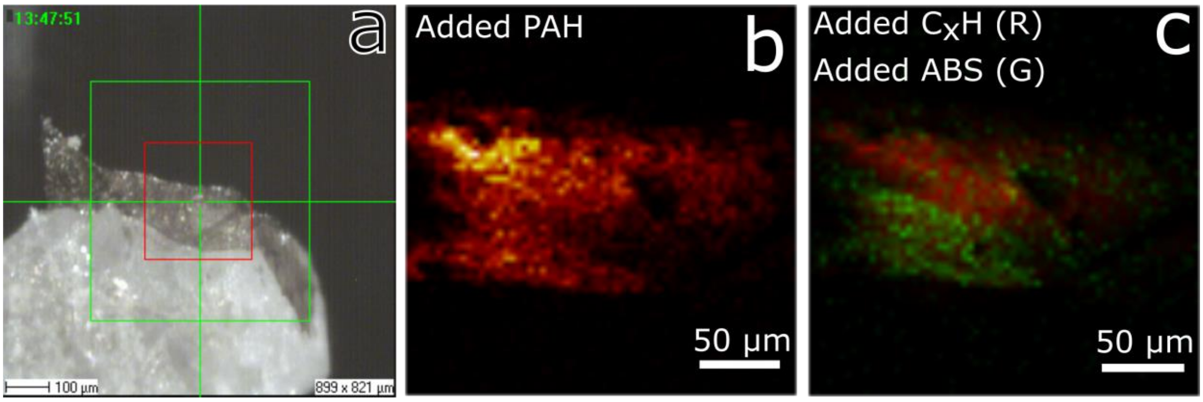

Supplementary Figure 4. ToF-SIMS ion images of putative organic material (**a**) ToF-SIMS video image of putative organic material on a carbonate crystal in sample CC1:537. Red square indicates area of ToF-SIMS analysis. (**b**) Combined positive ion image of PAHs at m/z 77.04 (C_6H_5) 91.05 (C_7H_7), 115.05 (C_9H_7), 128.05 ($C_{10}H_8$), 139.05 ($C_{11}H_7$), 141.06 ($C_{11}H_9$), 152.05 ($C_{12}H_8$), 165.06 ($C_{13}H_9$), 178.06 ($C_{14}H_{10}$), 189.05 ($C_{15}H_9$) and 202.06 ($C_{16}H_{10}$) (**c**) Negative ToF-SIMS ion image overlay of CxH peaks (red; added C_2H, C_4H, C_6H, C_8H, $C_{10}H$ and $C_{12}H$) and alkyl benzene sulfonates (ABS green; added $C_{27}H_{47}SO_3$, $C_{28}H_{49}SO_3$, $C_{29}H_{51}SO_3$, $C_{30}H_{53}SO_3$ and $C_{31}H_{55}SO_3$). The latter can be assigned to alkylbenzene sulfonates which are probably derived from drilling additives. The ion images of PAHs, C_xHs fragments (presumably produced by PAHs) and alkylbenzene sulfonates show that these compounds have different spatial distribution indicating two different sources for the PAHs and sulfonates (Supplementary Figure 3). Additionally, the PAHs have more typical geological peak pattern in the spectra with decreasing intensity with increasing mass compared with the alkyl benzene sulfonates. As water and not oil was used during drilling no additional PAHs should have been added to the system.

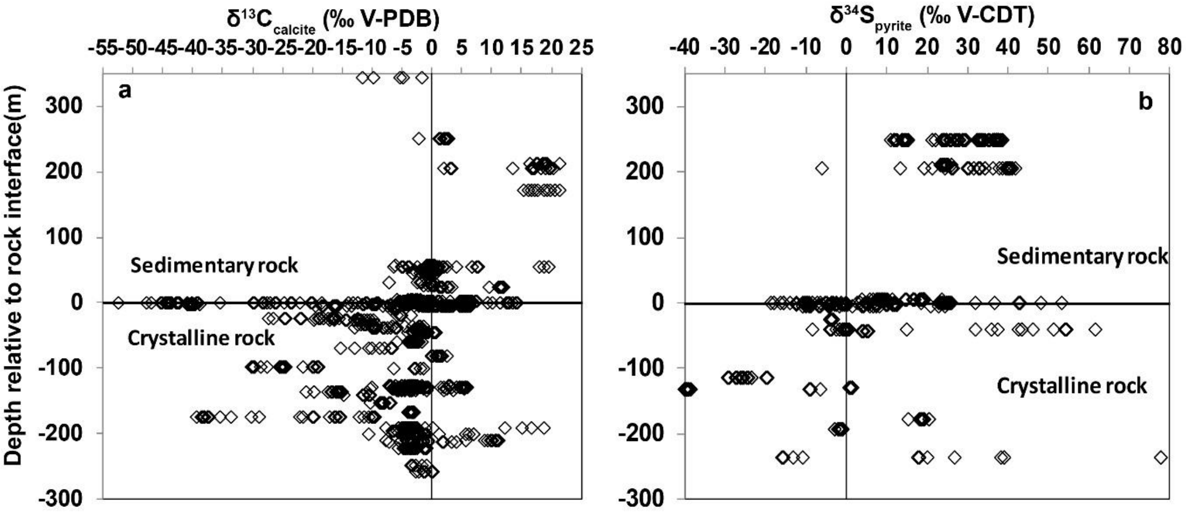

Supplementary Figure 5. $\delta^{13}C_{calcite}$ and $\delta^{34}S_{pyrite}$ versus depth. (**a**) $\delta^{13}C_{calcite}$, depth-normalized to sedimentary-crystalline rock interface. (**b**) $\delta^{34}S_{pyrite}$, depth-normalized to sedimentary-crystalline rock interface. Errors (2σ) are within the size of the symbols.

Supplementary references

1 Head, I. M., Jones, D. M., Larter SR. Biological activity in the deep subsurface and the origin of heavy oil. Nature. **426,** 344-352 (2003).

2 Pallasser, R. J. Recognising biodegradation in gas/oil accumulations through the $\delta^{13}C$ compositions of gas components. *Organic Geochemistry* **31**, 1363-1373 (2000).

3 Boreham, C. J., Edwards, D. S. Abundance and carbon isotopic composition of neo-pentane in Australian natural gases. Organic Geochemistry. **39**, 550-566 (2008).

4 Ahmed, M., Lehnert, O., Fuentes, D. & Meinhold, G. Origin of oil and bitumen in the Late Devonian Siljan impact structure, central Sweden. *Organic Geochemistry* **68**, 13-26 (2014).

5 Jeffrey, A. W. A. & Kaplan, I. R. Hydrocarbons and inorganic gases in the Gravberg-1 well, Siljan Ring, Sweden. *Chemical Geology* **71**, 237-255, (1988).

6 Smellie, J. A. T. & Tullborg, E.-L. Geochemical investigations in the Siljan area, Sweden. SGAB Internal Report, IRAP 85214. (SGAB, Göteborg/Uppsala, Sweden, 1985).

7 Röling, W. F. M., Head, I. M., Larter, S. R., The microbiology of hydrocarbon degradation in subsurface petroleum reservoirs: perspectives and prospects. *Research in Microbiology*. **154,** 321-328 (2003).

8 Elvert, M., A. Boetius, K. Knittel, and B. B. Jørgensen, Characterization of Specific Membrane Fatty Acids as Chemotaxonomic Markers for Sulfate-Reducing Bacteria Involved in Anaerobic Oxidation of Methane, *Geomicrobiology Journal*, **20**, 403-419. (2003)

9 Zheng, Y., Harris, D. F., Yu, Z., Fu, Y., Poudel, S., Ledbetter, R. N., et al. A pathway for biological methane production using bacterial iron-only nitrogenase. *Nature Microbiology*. **3**:281-6 (2018).

10 Guo, H., Zhang, J., Han, Q., Huang, Z., Urynowicz, M. A., Wang, F. Important Role of Fungi in the Production of Secondary Biogenic Coalbed Methane in China's Southern Qinshui Basin. *Energy & Fuels*. **31**:7197-207 (2017).

11 Lenhart, K., Bunge, M., Ratering, S., Neu, T. R., Schüttmann, I., Greule, M., et al. Evidence for methane production by saprotrophic fungi. *Nature Communications*. **3**:1046 (2012).

12 Sohlberg, E., Bomberg, M., Miettinen, H., Nyyssönen, M., Salavirta, H., Vikman, M., et al. Revealing the unexplored fungal communities in deep groundwater of crystalline bedrock fracture zones in Olkiluoto, Finland. *Front Microbiol.* **6**:573 (2015).

13 Drake. H., Ivarsson, M. The role of anaerobic fungi in fundamental biogeochemical cycles in the deep biosphere. *Fungal Biology Reviews*. **32**:20-5. (2018)

14 Lehnert, O., Meinhold, G., Wu, R., Calner, M., Joachimski, M. M. $\delta^{13}C$ chemostratigraphy in the upper Tremadocian through lower Katian (Ordovician) carbonate succession of the Siljan district, central Sweden. Estonian Journal of Earth Sciences. **63,** 277-286. (2014).

15 Drake, H., Tullborg, E-. L., Sandberg, B., Blomfeldt, T., Åström, M. E., Extreme fractionation and micro-scale variation of sulphur isotopes during bacterial sulphate reduction in Deep groundwater systems. Geochimica et Cosmochimica Acta. **161** (2015).

16 Drake, H. *et al.* Extreme ^{13}C-depletion of carbonates formed during oxidation of biogenic methane in fractured granite. *Nature Communications* **6**, 7020 (2015).

17 Etiope, G. & Sherwood Lollar, B. Abiotic methane on Earth. *Reviews of Geophysics* **51**, 276-299 (2013).

18 Milkov, A. V. & Etiope, G. Revised genetic diagrams for natural gases based on a global dataset of >20,000 samples. *Organic Geochemistry* **125**, 109-120 (2018).

19 Tazaz, A. M., Bebout, B. M., Kelley, C. A., Poole, J. & Chanton, J. P. Redefining the isotopic boundaries of biogenic methane: Methane from endoevaporites. *Icarus* **224**, 268-275 (2013).

20 Vlierboom, F. W., Collini, B. & Zumberge, J. E. The occurrence of petroleum in sedimentary rocks of the meteor impact crater at Lake Siljan, Sweden. *Organic Geochemistry* **10**, 153-161 (1986).

21 Kohn, M. J., Riciputi, L. R., Stakes, D. & Orange, D. L. Sulfur isotope variability in biogenic pyrite; reflections of heterogeneous bacterial colonization? *American Mineralogist* **83**, 1454-1468 (1998).

22 Parrish, R. R., Parrish, C. M. & Lasalle, S. Vein calcite dating reveals Pyrenean orogen as cause of Paleogene deformation in southern England. *Journal of the Geological Society* (2018).

23 Ring, U. & Gerdes, A. Kinematics of the Alpenrhein-Bodensee graben system in the Central Alps: Oligocene/Miocene transtension due to formation of the Western Alps arc. *Tectonics* **35**, 1367-1391 (2016).

24 Goodfellow, B. W., Viola, G., Bingen, B., Nuriel, P. & Kylander-Clark, A. R. C. Palaeocene faulting in SE Sweden from U–Pb dating of slickenfibre calcite. *Terra Nova* **29**, 321-328 (2017).
25 Pisapia, C. *et al.* U/Pb dating of geodic calcite: new insights on Western Europe major tectonic events and associated diagenetic fluids. *Journal of the Geological Society* (2017).
26 Roberts, N. W. & Walker, R. J. U-Pb geochronology of calcite mineralized faults; absolute dating of rift-related fault events on the northeast Atlantic margin. *Geology* **44**, 531-534 (2016).
27 Mortimore, R. N. Late Cretaceous tectono-sedimentary events in NW Europe. *Proceedings of the Geologists' Association* **129**, 392-420 (2018).
28 Japsen, P., Green, P. F., Bonow, J. M. & Erlström, M. Episodic burial and exhumation of the southern Baltic Shield: Epeirogenic uplifts during and after break-up of Pangaea. *Gondwana Research* **35**, 357-377 (2016).
29 Jourdan, F., Reimold, W. U. & Deutsch, A. Dating Terrestrial Impact Structures. *Elements* **8**, 49 (2012).
30 Siljeström, S. et al. Detection of organic biomarkers in crude oils using ToF-SIMS. *Organic Geochemistry* **40**, 135-143 (2009).
