# Supplementary material for: Timing and origin of natural gas accumulation in the Siljan impact structure, Sweden
Source: Nat Commun. 2019 Oct 18;10:4736. doi: 10.1038/s41467-019-12728-y (PMC6802084; doi:10.1038/s41467-019-12728-y)
Supplement: Supplementary file 3 — Description of Additional Supplementary Files [file 41467_2019_12728_MOESM3_ESM.pdf]

Description of Additional Supplementary Files

File Name: Supplementary Data 1
Description: SIMS data (δ13C, δ18O) and LA-MC-ICP-MS data (87Sr/86Sr) of calcite, including measurements of reference materials.

File Name: Supplementary Data 2
Description: SIMS data for δ34S in pyrite.

File Name: Supplementary Data 3
Description: Fluid inclusion data in fracture coating calcite.

File Name: Supplementary Data 4
Description: LA-ICP-MS U-Pb carbonate geochronology analytical data.

File Name: Supplementary Data 5
Description: Summary table for carbonate U-Pb geochronology.

File Name: Supplementary Data 6
Description: Analytical Conditions for U-Pb geochronology.

File Name: Supplementary Data 7
Description: n-Alkane and regular isoprenoid parameters in ground coating samples.

File Name: Supplementary Data 8
Description: Gas compositions from the boreholes.
